# Human chorionic gonadotropin elevation in gliomatosis peritonei complicated with immature teratoma: A case report and review of the literature

**DOI:** 10.1097/MD.0000000000031305

**Published:** 2022-10-28

**Authors:** Fei Guo, Yukai Liu, Jiaqi Lu, Zhiyong Wu, Xiaoyong Zhu

**Affiliations:** a Department of Gynecology, Obstetrics and Gynecology Hospital of Fudan University, Shanghai, China.

**Keywords:** chemotherapeutic retroconversion, gliomatosis peritonei, HCG elevation, ovarian immature cystic teratoma

## Abstract

Gliomatosis peritonei (GP) refers to the implantation of glial tissue on the visceral and parietal peritoneal surface, often associated with immature teratoma. It is a rare condition and the pathogenesis is not fully understood. In addition, the indistinguishable radiological appearance of immature and mature teratomas, and limited pathology samples make an accurate diagnosis difficult in most cases. More importantly, patients are also at risk of recurrence after surgery. This report aims to describe the process of diagnosis and treatment of GP with immature teratoma.

**Patient concerns:**

The patient, a 38-year-old woman presented with GP complicated with immature teratoma after laparoscopic ovarian cyst excision.

**Diagnoses:**

On physical examination, a 15 cm-pelvic mass, with poor mobility, was palpated. And tumor marker demonstrated a moderate increase in *α*-fetoprotein and carbohydrate antigen 125. We suspected malignancy according to the comprehensive preoperative evaluation, the postoperative pathology revealed an immature teratoma of the left ovary and complicated with gliomatosis peritonei. Three months after the second surgery, possible recurrence of immature teratoma was considered and the patient underwent the third laparotomy. But the postoperative pathology indicated mature teratoma and mature glial components in the pelvic lesions.

**Interventions and outcome:**

The patient underwent 2 more surgical resections after the initial resection and 3 cycles of bleomycin, etoposide, and cisplatin regimen chemotherapy. She was regularly followed up in the outpatient after surgery, and no recurrence has been reported in the pelvic cavity till date.

**Lesson:**

The case illuminated that the primary diagnosis of GP complicated with immature teratoma is critical but highly challenging for both gynecologists and pathologists and more attention should be paid to “GP complicated with immature cystic teratoma” patients to avoid inappropriate treatment.

## 1. Introduction

Gliomatosis peritonei (GP) refers to the implantation of glial tissue on the visceral and parietal peritoneal surface, often associated with immature cystic teratoma (IMCT). It is a rare condition, only around 130 cases recorded, with a favorable prognosis in most.^[1–4]^ Treatment is based on the stage and grade of the primary ovarian tumor since the residual peritoneal lesions in GP can be asymptomatic and quiescent over a long period.^[4]^ The pathogenesis of GP is not fully understood. It may correlate to defect of primary tumor capsular^[5]^ or capsular rupture during operation.^[6]^ In addition, genetic evidence has demonstrated they have unique genetic characteristics.^[7,8]^ The IMCT along with many serum tumor markers increased, for example, carbohydrate antigen 125 (CA125)^[4,9]^ and *α*-fetoprotein (AFP).^[10,11]^ However, no studies have reported that an elevated level of serum human chorionic gonadotrophin (HCG) is associated with IMCT or GP so far. Here, we present a case of GP complicated with ovarian IMCT after laparoscopic ovarian cyst excision and a review of relevant literature.

## 2. Case presentation

A 38-year-old female presented to our hospital with a recurrent pelvic cyst with no abdominal discomfort or vaginal bleeding following a laparoscopic left ovarian cyst excision performed 8 months ago due to a peduncular torsion of the ovarian cyst in another institution. The patient was G3P1 with a regular menstruation cycle. Her family history is unremarkable. A 15 cm-pelvic mass, with poor mobility, was palpated during the gynecological examination. The magnetic resonance imaging suggested there were bilateral ovarian teratomas (right for 126 × 119 × 82 mm, left for 83 × 81 × 78 mm), and the analysis of tumor marker levels demonstrated a moderate increase in AFP (29.10 ng/mL; normal range < 9 ng/mL) and CA125 (125.70 U/mL; normal range < 35 U/mL). The other tumor marker levels were in the normal range.

**Figure 1.**
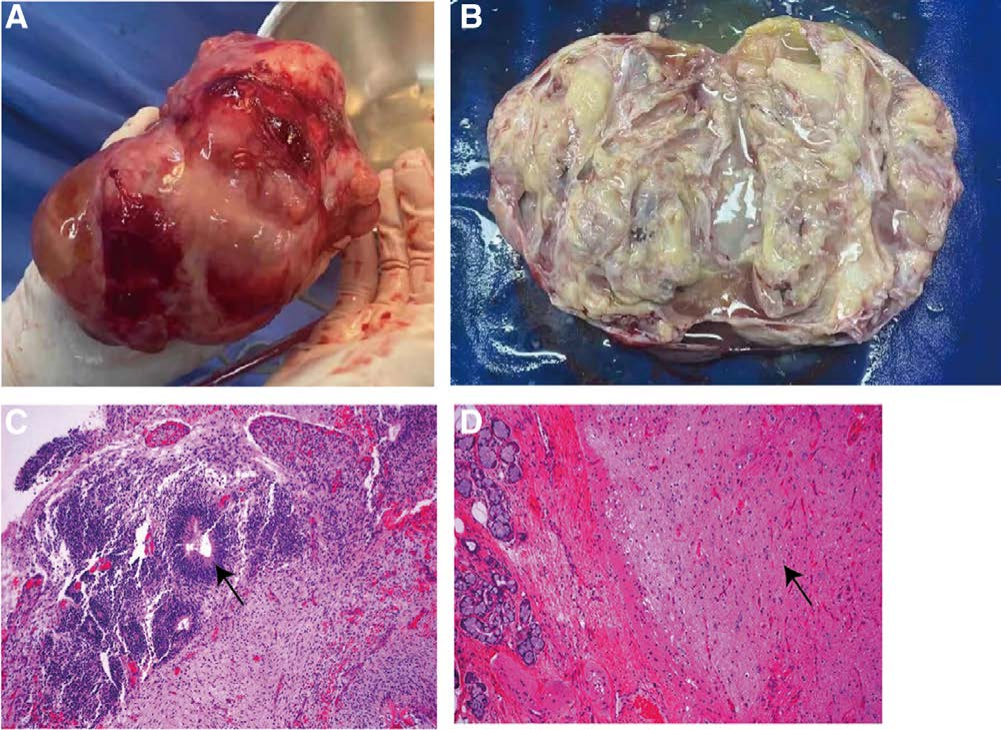
All the pictures from the second laparoscopy. (A) Anterior view the immature teratoma of the left ovary; (B) Section view of the immature teratoma of the left ovary; (C) Neural tube component from the immature teratoma of left ovary H&E, X 10; (D) Mature nerve tissue (right) and respiratory gland (left) from the peritoneal lesion H&E, X 10. “→” represents where lesions are located.

We suspected malignancy according to the comprehensive preoperative evaluation. The second laparotomy was operated on December 24, 2020. During the operation, we found that the left ovary was cystic and solid, irregularly enlarged with a diameter of about 10 cm. The left fallopian tube adhered tightly to the surface of the left ovary. Another mass of about 18 cm was observed on the surface of sigmoid colon. There was no abnormality on the right ovary and fallopian tube. The left ovarian cyst was removed and the intraoperative frozen section was suggestive of mature teratoma with extensive implantation of glial components in the pelvic and abdominal cavity. According to the National Comprehensive Cancer Network guideline, cystectomy was preferred given the benign nature of mature teratomas. However, considering the risk of recurrence with the widespread implantation of glial components, and possible postoperative histopathology indication of malignancy, we performed left salpingo-ovariectomy and pelvic lesion resection. The postoperative pathology revealed, a grade II immature teratoma of the left ovary, no lesion on the left fallopian tube, Other lesions presented with mature teratoma components and complicated with a large number of mature glial implants (Fig. 1). As per the National Comprehensive Cancer Network guideline, the patient intended to receive 6 cycles of intravenous chemotherapy using the bleomycin, etoposide, and cisplatin regimen (bleomycin, etoposide, and cisplatin). Due to the severe side effects of chemotherapy, especially nausea and vomiting, the patient refused to comply with the remaining 3 cycles.

**Figure 2.**
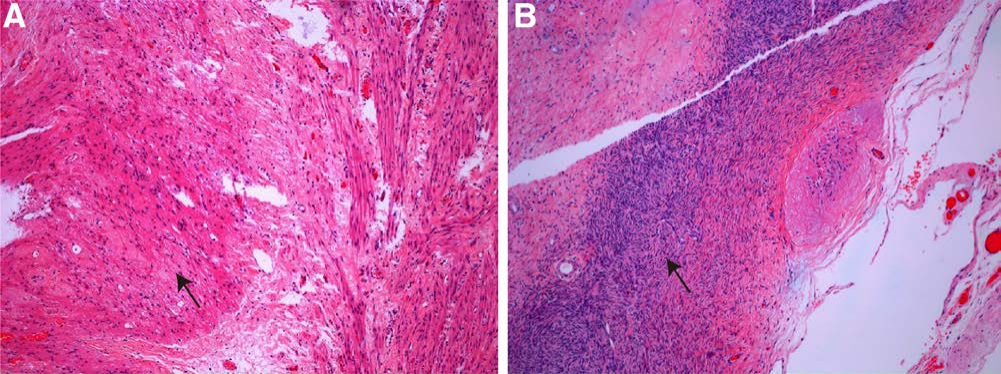
All the pictures from the third laparotomy. (A) Glial component from the serosal surface of the uterine H&E, X 10; (B) Glia component from the surface of the right ovary H&E, X 10. “→” represents where lesions are located.

**Figure 3.**
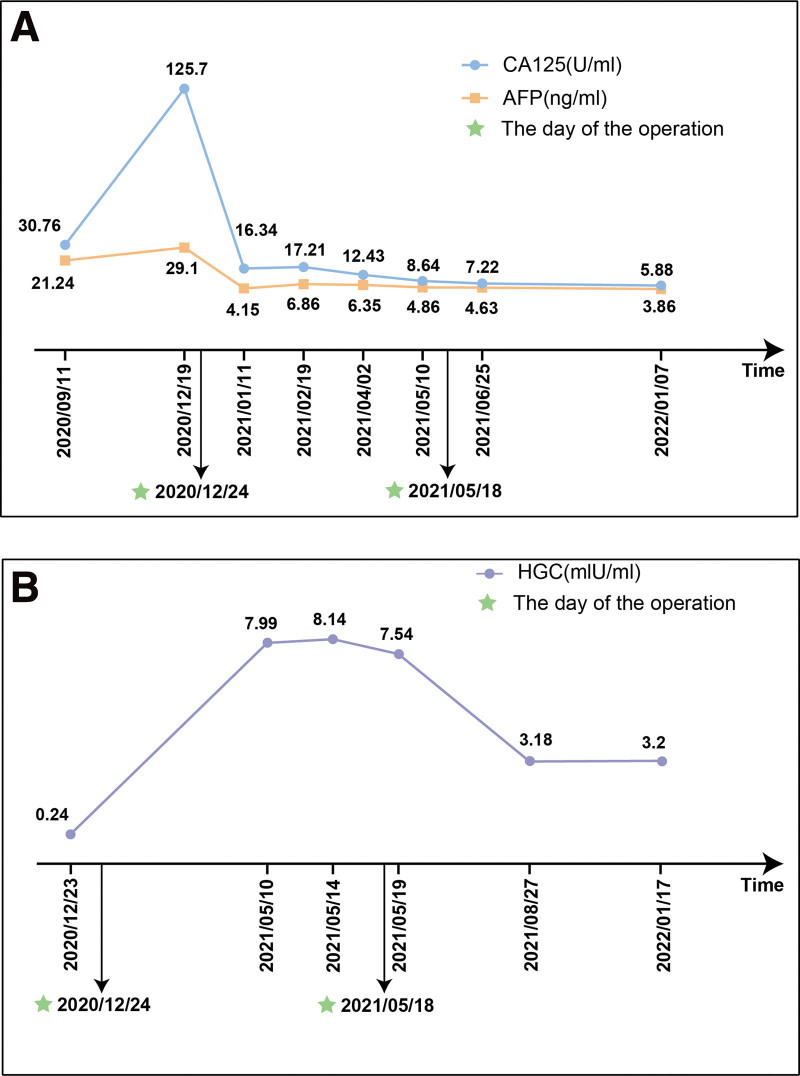
(A) The CA125 and AFP levels fluctuation during the treatment. (B) The HCG level fluctuation during the treatment. “*” represents the date of laparotomy.

Frustratedly, a month after the operation, the B-ultrasound revealed a mass about 18 × 16 × 8 mm in the right ovary, and an irregularly shaped subcutaneous mass measuring 30 × 21 × 14 mm in the left lower quadrant abdomen. Three months later, the pelvic mass increased to 70 × 50 × 48 mm, and the subcutaneous mass increased to 47 × 41 × 17 mm. The levels of CA125 and AFP remained in the normal range. However, the serum HCG level elevated to 8.14 mIU/mL (normal range < 5 U/mL). Considering the rapid expansion of the lesion in the short time and possible recurrence of immature teratoma, the patient underwent the third laparotomy with a total hysterectomy, right adnexectomy, and pelvic lesion resection. The postoperative pathology indicated mature teratoma in the left paracolic sulcus lesions and abdominal wall lesions, and mature glial components in the pelvic lesions (Fig. 2). The patient was regularly followed up in the outpatient after surgery, and no abnormalities have been found so far (Fig. 3).

## 3. Discussion and review of the literature

### 3.1. Immature cystic teratoma

Ovarian teratoma is a common ovarian lesion (15%–20% of all cystic ovarian tumors),^[12]^ including mature and immature tissue of germ cell origin. Mature cystic teratoma (MCT) is the most common subtype of ovarian teratomas, commonly termed “dermoid cyst”, which usually contains mature tissues of the ectoderm (skin, brain), mesoderm (muscle, fat), and endoderm (sticky or ciliated epithelium). The probability of the malignant transformation of MCT usually does not exceed 1% to 2%.^[13]^ Similarly, the tissues of IMCT can be traced to the 3 embryonic germ cells, especially the ectoderm, with the most abundant tissue component being neurogenic in origin.^[14]^ In general, IMCT possesses larger tumor volume and higher malignancy potential when compared to MCT.^[15]^ Moreover, it has a unique characteristic called chemotherapeutic retroconversion (CR), where its malignant degree is reversed.^[16]^ The phenomenon of CR is considered to occur infrequently previously.^[17,18]^ Nevertheless, CR seems to be more and more common according to the recent research.^[10,19]^ As reported in our case, the ovarian immature teratoma also reported mature teratoma in the third laparotomy after 3 cycles of chemotherapy.

Nowadays, the laparoscopic removal of ovarian cysts is a state-of-the-art procedure. However, the cyst rupture with intraperitoneal spillage occurs in 18% to 57.8% of laparoscopy compared with laparotomy.^[20,21]^ Effects of an unintended rupture of IMCT are prognostically relevant because they change a possible stage 1A to stage 1C.^[22]^ The rupture of MCT also leads to acute peritonitis as Rubod reported.^[23]^ The remission rates of malignant ovarian germ cell tumor have increased dramatically with the introduction of platinum-based chemotherapy.^[24]^ The 5-year survival rates have increased to 99% and 69% in the stage I patients and stage IV patients with distant metastasis.^[25]^ No long-term adverse effects on reproductive function have been observed in the fertility-preserving patients.^[26,27]^ However, approximately 20% of patients are resistant to chemotherapy or relapse after surgery.^[28]^

### 3.2. Gliomatosis peritonei

#### 3.2.1. Implant mechanisms.

Histologically, the implants of GP are similar to the benign mature glial tissue, with delicate fibrous processes and scattered supporting cells. There are 2 theories about the biogenesis of peritoneal gliomatosis. One theory suggests that glial implantation of GP comes from the differentiation of peritoneal pluripotent mullerian stem cells.^[29]^ The tumor contains a large amount of glia and secretes growth factors to stimulate peritoneal stem cells to differentiate into glial cells. Nogale also supports that peritoneal stem cell differentiation could explain the single form of astrocytes presented by GP in some cases.^[30]^ Another theory proposes that the glia is differentiated from tumor cells in the ovarian immature teratoma.^[3,31]^ The macrophage-derived factors could allow the neural component of ovarian teratoma to implant in the peritoneal cavity and induce astroglial differentiation, which could explain why the peritoneal implants are mostly mature even when they originate from immature teratomas.^[32]^ The detailed information of peritoneal gliomatosis remain to be determined.

#### 3.2.2. Diagnosis.

The diagnosis of GP depends chiefly on pathological sections, but the inflammatory cell infiltration, hemorrhage, and choroid plexus can also be seen in the sections, which makes it difficult to diagnose, especially the intraoperative frozen sections. In these challenging situations, immunohistochemical staining for glial fibrillary acidic protein and S100 protein may be helpful.^[14]^ Moreover, Li demonstrated that the tumor cells expressed sex determining region Y-box 2 but not octamerbinding transcription factor 4 or homeobox protein Nanog homeobox in all GP specimens in their cases.^[3]^

#### 3.2.3. Prognosis and treatment.

The mature glial cells are noninvasive, the implantation could remain stable for a long time and the malignant transformation is extremely rare.^[3,33]^ There is no difference in overall survival between IMCT with GP or without GP, though patients with GP present with more frequent recurrence and shorter recurrence-free survival^[34]^ (Table 1). And Robboy discovered that the prognosis was favorable when the peritoneal implants were composed of fully mature glial tissue.^[37]^ Therefore, many researchers suggest we do not have to resect the tumor in asymptomatic patients.^[34,38]^

**Table 1:** Summary of reports of GP complicated with teratoma.

Reference	Number of cases	Median follow-up time	Prognosis
Wang et al^[4]^	8	60.5 mo	All alive and asymptomatic
Yoon et al^[34]^	15	2547 d	1 patients died
Liang et al^[3]^	21	23 mo	5 were lost to follow-up, 13 were alive with no disease, 3 were alive with disease
Małgorzata et al^[35]^	1	5 yr	Alive and remission
Wang et al^[6]^	2	4 yr	Alive and without recurrence or metastasis
Ohara et al^[36]^	1	1 yr	Alive and without recurrence

In our case, the GP lesions recurred 1 month after the operation and it continued to grow up after 3 cycles of chemotherapy. Although chemotherapy achieves the retroconversion of IMCT to MCT, whether it is an effective treatment for GP is questionable. Harms pointed out that when GP included immature implants, additional chemotherapy can induce maturation of the implants.^[39]^ Furthermore, the GP lesions are extensive, and complete excision in 1 go is usually difficult. Therefore, it requires a careful monitoring of residual lesions using scanning imaging such as computerized tomography and magnetic resonance imaging, which are safe, reproducible, and accurate techniques for patients who are associated with GP.^[14]^

### 3.3. HCG elevation

We accidentally discovered that the level of HCG increased significantly to 8.14 mIU/mL and a slight decline was noted after resecting the recurrent tumor in the third laparotomy. Three months later, the HCG level decreased to the normal range. A head computerized tomography was performed on this patient to rule out abnormality in pituitary. There were no studies have reported GP with IMCT accompanied by an elevated level of HCG so far, but Downey reported a case of ectopic HCG secretion by a mature teratoma.^[40]^ In addition to the secretion from the pituitary, we propose 2 hypotheses: On the 1 hand, the elevated HCG level is derived from the glial components of GP. With the stabilization of glial components, the level of HCG decreases gradually. On the other hand, HCG is secreted by mature teratoma reversed from immature teratoma after chemotherapy. Whether HCG is a tumor marker for GP is still uncertain, but we should pay more attention to the HCG levels in these groups. Further cases and studies are required to support the role of serum HCG as a predictor of GP.

## 4. Conclusions

In our case, GP complicated with IMCT was treated with 3 surgeries and 3 cycles of bleomycin, etoposide, and cisplatin regimen chemotherapy. We summarize some of the lessons learned from this case. First, we advocate that content spillage should be avoided during the operation, especially when the cyst is suspected to be malignant. When removing the cyst, we should place it into a surgical retrieval apparatus and appropriately extend the incision during operation. In case of inevitable content spillage, complete suction of the spilled contents and thorough rinse of the abdominal cavity with physiological saline to avoid implantation. Second, the radiological features of IMCT and MCT are indistinguishable, and the samples from intraoperative frozen sections are limited. It undoubtedly challenges the clinical diagnosis and operation scheme. Hence, multiple intraoperative and postoperative samples of the lesions must be obtained and careful analysis of tissue sections must be carried out to make accurate judgments. In addition, after exclusion of the presence of immature elements in massive peritoneal lesions, a more conservative treatment may be considered for young women who wish to preserve fertility.^[4,41]^ However, the volume of GP becomes large or symptoms develop in the short term, it is recommended to be resected. Finally, GP is often accompanied by numerous peritoneal implants, and complete resection is difficult to achieve. Therefore, the frequency of postoperative follow-up should increase appropriately. It is recommended every 3 months for the first 2 years after surgery, every 6 months during years 3 through 5, then once a year thereafter, with the psychological preparation to undergo multiple surgeries.

## Authors’ contributions

**Conceptualization:** Fei Guo.

**Data curation:** Yukai Liu, Jiaqi Lu.

**Formal analysis:** Jiaqi Lu.

**Funding acquisition:** Xiaoyong Zhu.

**Investigation:** Fei Guo.

**Methodology:** Yukai Liu.

**Resources:** Zhiyong Wu.

**Supervision:** Xiaoyong Zhu.

**Validation:** Zhiyong Wu.

**Writing – original draft:** Fei Guo.

**Writing – review & editing:** Xiaoyong Zhu.

## Correction

The funding number 2071624 in the footnote has been corrected to 82071624.
